# Bevacizumab and CCR2 Inhibitor Nanoparticles Induce Cytotoxicity-Mediated Apoptosis in Doxorubicin-Treated Hepatic and Non-Small Lung Cancer Cells

**DOI:** 10.31557/APJCP.2019.20.7.2225

**Published:** 2019

**Authors:** Ahmed A Abd-Rabou, Hanaa H Ahmed

**Affiliations:** 1 *Hormones Department, Medical Research Division,*; 2 *Stem Cell Laboratory, Center of Excellence for Advanced Science, National Research Centre, Giza, Egypt. *

**Keywords:** Bevacizumab (avastin)- CCR2 antagonist- non-small cell lung cancer- hepatocellular carcinoma- cytotoxicity

## Abstract

Non-small cell lung cancer (NSCLC) and hepatocellular carcinoma (HCC) are very common in certain population around the world. Despite the recent advances in their diagnosis and therapy, their prognosis remains poor due to the development resistance to drug. Although doxorubicin (DOX) is considered to be one of the most anti-solid tumor drugs, developed resistance is contributing to unsuccessful outcome. The rationale of the current study is to explore the sensitizing capability of the DOX-treated cancer cells using the anticancer agents; bevacizumab (avastin; AV) and CCR2 inhibitor (CR) in their free- and nano-formulations. Here, the average size, polydispersity index (PDI), zeta potential, and entrpment effeciency (EE%) of the synthesized nanoparticles were measured. We investigated the effect of these platforms on the proliferation, apoptosis, necrosis, nitric oxide (NO), malondialdehyde (MDA), and zinc levels of human HCC (HepG2 and Huh-7) and NSCLC (A549) cancer cell lines. Glucose consumption rates using Huh-7 and A549 cancer cells were tested upon treatments. We demonstrated that AV and CR nano-treatments significantly suppressed A549 cell viability and activated apoptosis by NO level elevation. We concluded that AVCR NP plus DOX significantly induces A549 cytotoxicity-mediated apoptosis more than Huh-7 and HepG2 cells. This drug-drug nano-combination induced Huh-7 cytotoxicity-mediated apoptosis more than HepG2 cells. In conclusion, AVCR NP sensitized DOX-treated A549 and Huh-7 cells through reactive oxygen species (ROS)-stimulated apoptosis. Taken together, our data suggested that the CR plus AV nano-platforms would be a potential personalized medicine-based strategy for treating CCR2-positive NSCLC and HCC patients in the near future.

## Introduction

Cancer, as a multifactorial aliment, is a chief cause of mortality globally. Hepatocellular carcinoma (HCC) and non-small cell lung cancers (NSCLC) are examples of such epidemic aliment (Wu et al., 2011). HCC represents one of the leading causes of mortality worldwide (Abd-Rabou and Ahmed, 2017; Siege et al., 2017). HCC accounts for 854 thousand incident cases and 810 thousand deaths globally (Global Burden of Disease Cancer Collaboration, 2017). NSCLC, A549 cell line as an example, is the most common type of lung cancer, which is the leading cancer killer worldwide (Goldstraw et al., 2011). Cancer patients of this specific type can be classified into three categories: early, locally advanced, and distant metastasis. Unfortunately, the prognosis of those patients remains unsuccessful, despite the recent advances in anticancer therapies, perhaps owing to late diagnosis until advanced or metastatic stages happened (Yang, 2009).

Although the presence of different chemotherapeutic approaches for tackling HCC and NSCLC, drug resistance is still a remaining obstacle that finally ends up with cancer relapse. Hence, some missing acquaintances are present between the fundamental carcinogenic machineries and the current plans of drug development (Lynch et al., 2004; Shivakumar et al., 2016; Sasaki et al., 2011; Soucek et al., 2008; Rosell and Felip, 2001; Wu et al., 2011). Therefore, there is an urgent need for new therapeutic approaches for HCC and NSCLC.

Doxorubicin (DOX) is an important drug in many chemotherapy regimens. Although DOX is presently considered to be one of the most active agents in the treatment of solid cancers, resistance leads to an unsuccessful outcome in many circumstances (Smith et al., 2006), leading to up-regulation of the expressions of anti-apoptotic genes and activated intracellular survival signal following cellular stress (Xue and Liang, 2012). Production of the cellular energy through the oxidative phosphorylation and mitochondrial respiration is essential for cancer progression. Moreover, mitochondria control the production of reactive oxygen species (ROS) and in turn the cellular apoptosis. Intriguingly, mitochondria play an important role in cancer metabolic and apoptotic regulation via generation of ROS (Księżakowska-Łakoma et al., 2014; Zhong and Oberley, 2001). 

Chemokines are a superfamily plays with their receptors in many pathological procedures like cancer (Conti and Rollins, 2004; Fang et al., 2012). One of these chemokines is chemokine (C-C motif) ligand 2 (CCL2) which is also known as monocyte chemotactic protein-1 (MCP-1). In 1989, it was reported that CCL2 participates in monocytes recruitment during angiogenesis (Salcedo et al., 2000; Tangirala et al., 1997; Zachariae et al., 1990). CCL2 is produced by a variety of activating cells, such as lymphocytes and macrophages (Zachariae et al., 1990) . Recent studies have reported that CCL2 is overexpressed in a majority of solid cancer types, including gastrointestinal cancers (Monti et al., 2003; Wolf et al., 2012; Zhang et al., 2010) and NSCLC (Zhang et al., 2013). 

Importantly, CCL2, which secreted by many cancer cells facilitates cancer metastasis and blocks CCL2-CCR2 signaling by specific inhibitors augments CD8+ T-cell-mediated responses and inhibits the metastatic process (Fridlender et al., 2010; Qian et al., 2011). 

However, angiogenesis is the common leading cause of cancer progression, targeting the VEGF is still tricky. According to certain observations from human cancer studies, anti-VEGF therapy usually results in cancer elimination or regrowth in some cases, so it is a debatable aspect (Bottsford-Miller et al.,2012; Chen et al., 2016), thus combing an anti-VEGF antibody (bevacizumab= avastin= AV (Ferrara et al., 2005)) with CCR2 antagonist (CR) as a novel approach in the current study may provide new promising therapeutic window. 

In the current study, we have hypothesized to deliver AV and CR using nanotechnology to increase the bio-availability and stability of the synthesized nano-capsules and the targetability against cancer cells, as well as reduce the drug half inhibitory effect (IC_50_), thus decrease the cytotoxic effect on healthy cells (Chae et al., 2010). Many delivery strategies were developed by utilizing PEGylation and polymeric complexes for in vitro (Abd-Rabou al., 2018a; Abd-Rabou al., 2018b; Abd-Rabou al., 2018c; Abd-Rabou al., 2016; Shalby et al., 2017) and in vivo (Ahmed et al., 2018; Mohamed et al., 2018) applications. 

There are some nano-systems showed promise as drug delivery ones owing to their controlled and sustained release properties, subcellular size and biocompatibility with tissues and cells (Abd-Rabou al., 2018a; Abd-Rabou al., 2016). For AV, poly (DL-lactide-co-glycolide (PLGA) nanoparticles was used for its delivery with an entrapment efficiency (EE = 45%). The release of the encapsulated AV from the nanoparticles could last four weeks life-time (Hao et al., 2009). Pan and his companions also created long-lasting formulations (eight weeks) of AV through poly ethylene glycol (PEG)-coated PLGA nanoparticles (Pan et al., 2011). For CR, liposome targeted nano-delivery was approached to stimulate pulmonary endothelium and prevent metastasis (Roblek et al., 2015).

Generally speaking, the rationale of the present study is to design a novel anticancer therapy using bevacizumab (avastin; AV) and CCR2 inhibitor (CR)-encapsulated nanoparticles and applied them against hepatocellular carcinoma (HepG2 and Huh-7) and non-small cell lung cancer (A549) cell lines. The cytotoxicity-mediated apoptosis triggered by the redox status of these cell lines was mechanistically studied.

## Materials and Methods

Materials

Methoxy polyethylene glycol amine (mPEG-NH2, MW 5000 Da), 1-ethyl-3-(3-dimethylaminopropyl)-carbodiimide (EDC), N-hydroxysuccinimide (NHS), heparin, polyethylene glycol (MW 5000 Da), 2-(N morpholine) ethanesulfonic acid (MES), dimethyl sulfoxide (DMSO), Tween 80, poly L-lysine (PLL), CCR2 antagonist RS 504393 (CR), and glucose were purchased from (Sigma-Aldrich, USA). Bevacizumab avastin (AV) was purchased from (Genentech Inc., USA). Dulbecco’s modified Eagle’s medium (DMEM), Roswell Park Memorial Institute medium (RPMI-1640), fetal bovine serum (FBS), phosphate buffer saline (PBS), and Annexin V/propidium iodide (PI) apoptosis kit were purchased from (ThermoFisher Scientific, USA). Glucose detection kit and Zinc colorimetric assay kit were purchased from (Spectrum Diagnostics, Egypt). Lipid peroxidation colorimetric assay kit (MDA) and nitric oxid (NO) assay colorimetric kit were purchased from (Abcam, Cambridge, MA, USA). Ultrapure water (Millipore, Bedford, MA, USA) was used. 

*Preparation of AV and CR nanoparticles*

*Nano-void (NV) synthesis*

According to a previous method mentioned in (Lim et al., 2011), we used it with some modifications to prepare PEG-exposed nanoparticles (nano-void; NV). To prepare NV, we firstly prepared the mixture of EDC and NHS. Briefly, heparin (HP: 0.1 mMol) was coupled with 0.1 mM (formulation 1; F1), 0.2 mM (formulation 2; F2), or 0.3 mM (formulation 3; F3) of mPEG-NH2 (MW 5000 Da) using EDC (1.5 mMol) and NHS (1.6 mMol) in MES buffer (0.1 M, pH 5.5) at room temperature for 24 h stirring. Then, NV was prepared simply by mixing the polymer (PEG-heparin) with poly-L lysine buffer (PLL) overnight at 4^o^C. The mix ratio (1: 6 v/v, PLL: PEG-heparin). We used F2 to encapsulate AV and CR latter due to its better nano-size and particle stability as it will be mentioned in the results section. 

*Avastin nanoparticles (AV NPs) synthesis*

To prepare the PEG-nanoparticles of Bevacizumab avastin monoclonal antibody (AV NPs), firstly the PEG-heparin polymer was synthesized as above and the core nanoparticles were prepared by mixing the polymer with PLL buffer overnight at 4^o^C using this ratio (1:6 v/v, PLL: PEG-heparin). Then, AV NPs were synthesized by activating the amine groups in the formed nanoparticles mixture using EDC and NHS to bind with the carboxylic groups in 25 mg/mL monoclonal antibody (AV) by stirring overnight at 4oC, after that amide bonds were formed and the AV NPs were functionalized.

*CCR2 antagonist nanoparticles (CR NPs) synthesis*

To prepare the PEG-nanoparticles of CCR2 antagonist RS 504393 (CR NPs), simply the PEG-heparin polymer was synthesized as above and the CR nanoparticles were prepared by mixing the polymer with PLL buffer overnight at 4oC using this ratio (1: 6 v/v, PLL: PEG-heparin). Before adding PLL to form the CR NPs, 2 mg/mL CCR2 antagonist RS 504393 of CR was titrated to the PEG/heparin polymer. This mixture was vortexed for 5 min and subsequently sonicated for 5 min using a Sonics Vibra-cell sonifier VC750 equipped with a micro-tip (Newtown, CT) at amplitude = 35%, pulse-on = 5.0 s, and pulse-off = 3.0 s. The suspension was transferred to a round-bottom tube in a water bath with magnetic stirring overnight at room temperature.

*AVCR nanoparticles (AVCR NPs) synthesis*

To prepare AV-CR NPs, firstly the CR NPs were synthesized as a core vehicle (as mentioned above) then the amine groups were activated using EDC and NHS to bind the carboxylic groups in 25 mg/mL monoclonal antibody (AV) by stirring overnight at 4oC, after that amide bonds were formed and the AV-CR NPs were functionalized.

*NPs characterization*

*Dialysis technique and measurement of the encapsulation efficiency*

All nano-samples were divided into two portions; (1) one for measuring the total concentrations of the free plus nano-conjugated AV and CR, (2) the second part for measuring the concentrations of the nano-conjugated AV and CR after performing dialysis tubing technique for eliminating the impurities and the free drug using a membrane bag (Spectra/Por Membrane, Spectrum Laboratories, USA; molecular weight cut-off, MWCO: 25,000 Da).

The free and conjugated forms of AV and CCR2 antagonist were detected with a variable wavelength detector using UV-based ELISA system. The calibration curves for quantification of these compounds were linear over the range of standard concentrations. Finally, encapsulating (entrapment) efficiency (EE) was calculated after dialysis technology.

*Particle size distribution and zeta potential*

The particle size and zeta potential analyses of all nanoparticles were performed by photon correlation spectroscopy (PCS) and laser diffractometry (LD). For PCS measurements, 1mL of the nanoparticles solution was filled in the disposable transparent sizing clear cuvette and the size of sample was analyzed at 25^o^C with a Malvern ZetaSizer (Malvern Instruments, Westborough, Massachusetts). All measurements were performed in triplicate.

*In-vitro studies*

*Cell culture and maintenance*

Human hepatocellular carcinoma cell lines (HepG2 and Huh-7) and non-small cell lung cancer (A549) were purchased from VASCERA Co. (Vaccines Sera and Drugs) supplied them from American Type Culture Collection (ATCC, USA). Lung and liver cells were cultured using Dulbecco’s modified Eagle’s medium (DMEM) and Roswell Park Memorial Institute (RPMI-1640) medium. All media were supplemented with 4.5 g/L Glucose with L-Glutamine and 10% fetal bovine serum (FBS). The cells were incubated in 5% CO2 humidified at 37°C for growth maintenance.

*Measurement of cytotoxicity*

All drug groups were evaluated by MTT assay (van Meerloo et al., 2011) using HepG2, Huh-7, and A549 cancer cells. Briefly, the cells were cultured in 96-well plates at a density of 1×10^4^ cells/well. All AV and CR-based drugs with their described concentrations (0, 25, 50, 75, and 100 µM for AV and DOX, and nM for CR). were added in the media over these cell lines. Culture media with nano-void (i.e. nano-capsule without loaded drug) and without were added as controls for the drug-loaded nano-formulations and their free counterparts. After 24 h incubation, MTT dissolved in PBS was added to each well at a final concentration of 5 mg/ml, and the samples were incubated at 37°C for 4 h. Water-insoluble dark blue formazan crystals that formed during MTT cleavage in actively metabolizing cells were then dissolved in dimethyl sulfoxide (DMSO). Absorbance was measured at A540 nm, using a microplate reader (BMG Labtech, Germany). The cell viability (%) was calculated and compared with the controls. 

*Apoptosis measurement*

Annexin V/PI stains were used in determination of apoptosis (early and late) as well as necrosis after treatment with the nano-formulations and their free counterparts at 24 h of drug incubation with in HepG2, Huh-7, and A549 cancer cells. The apoptotic analysis was dedicated to differentiate between early and late apoptotic cells, as well as necrotic cells. The apoptosis of the treated and untreated HepG2, Huh-7, and A549 cancer cells with the IC_50_ dose for the drugs which recorded IC_50_ or 100 µM for DOX was analyzed by flow cytometer instrument (Beckman Coulter, USA).

*Biochemical measurements*

We selected Huh-7 cancer cells as a model for hepatocellular carcinoma to test the glucose consumption level and the redox status, because it was much sensitive against the used therapeutic regimens compared to HepG2 liver cancer cells. The glucose consumption level and the redox status (nitric oxide; NO, malondialdehyde; MDA, and zinc) of Huh-7 liver cancer cells versus A549 lung cancer cells were tested upon 0, 25, and 100 (µM for AV and DOX, and nM for CR) of AV, CR, AVNP, CRNP, and DOX+AVCRNP treatments. 

*Glucose consumption rate measurement*

Glucose consumption level using Huh-7 and A549 cancer cells were measured upon different nano-treatments and their free counterparts using glucose detection kit. 

Briefly, the cells were cultured in 96-well plates at a density of 1×10^4^ cells/well. In the second day, 5 mM of glucose and different nano-treatments and their free counterparts were added in the media after 2 hours of cells starvation. Absorbance was measured at A450 nm using the microplate reader (BMG Labtech, Germany) after 10 min incubation with the glucose detector. The glucose levels in mmol/L was calculated and compared with the untreated and nano-void controls.

**Figure 1:**
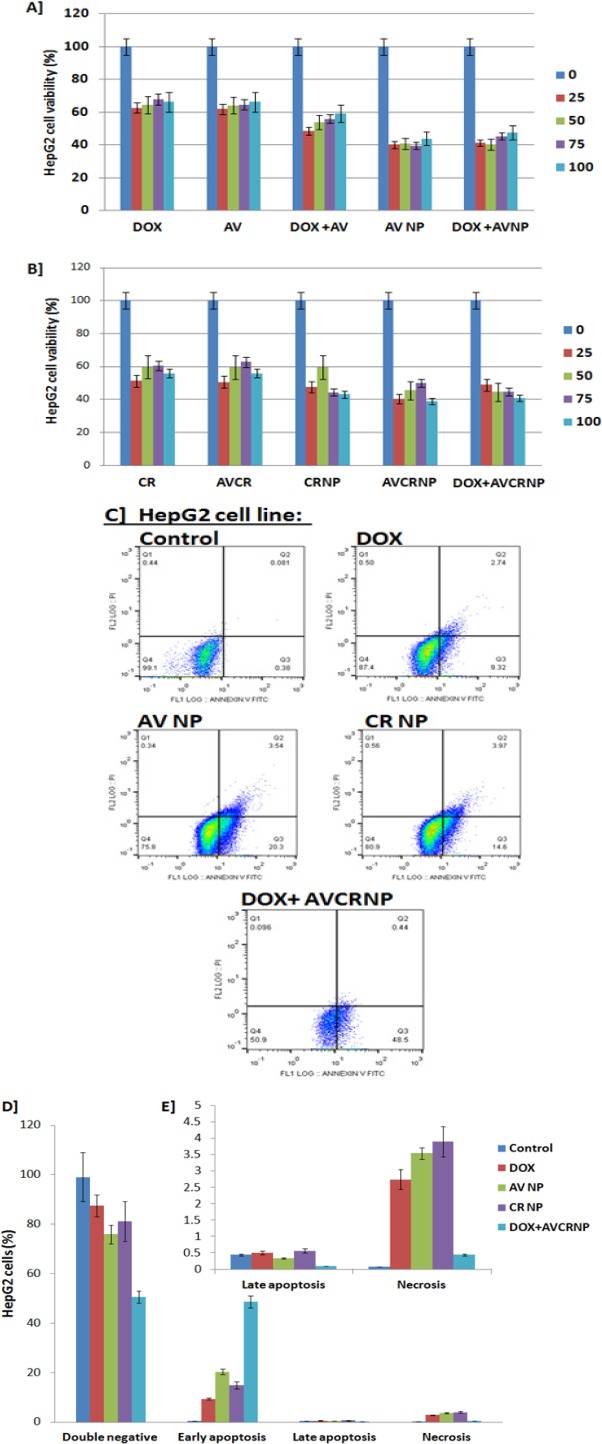
The Cytotoxic and Apoptotic Effects of the AV and CR Therapeutic Regimens against HepG2 Cancer Cell Line. A, HepG2 cell viability (%) upon DOX, AV, DOX+AV, AVNP, and DOX+AVNP treatments (n=3); B, HepG2 cell viability (%) upon CR, AVCR, CRNP, AVCRNP, and DOX+AVCRNP treatments (n=3). The doses used for cytotoxicity were 0, 25, 50, 75, and 100 (µM for AV and DOX, and nM for CR); C, Representative images of HepG2 cell apoptosis (annexin versus PI) using flow cytometry upon DOX, AVNP, CRNP, and DOX+AVCRNP treatments; D, E, Mean and standard error (n=3) of HepG2 cancer cell early and late apoptosis, as well as necrosis versus control upon DOX, AVNP, CRNP, and DOX+AVCRNP treatments. The used dose for the flow cytometry experiments was either the IC_50_ dose for the drugs which recorded IC_50_ or 100 µM for DOX.The cells were incubated with the drugs for 24 h

**Table 1:** Average of Size, PDI, Zeta Potential, and EE of the Different Synthesized Nanoparticles

NPs type	Formulations	Size (nm)	PDI	Zeta (mV)	EE (%)
NV	F1	108.9 ±5.37	0.1 ±0.00	4.99 ±0.27	-
	F2	110.3 ±2.04	0.04 ±0.00	-15.8 ± 2.10	-
	F3	116.3 ±6.5	0.1 ±0.01	15.3 ±5.04	-
AV NPs	F2	147.8 ±3.4	0.01 ±0.00	-9.05 ± 1.2	86
CR NPs	F2	76.26 ±3.4	0.07±0.00	-2.81±1.2	74
AV-CR NPs	F2	293.4 ±9.17	0.04±0.00	4.71 ±2.5	82 - 75

**Table 2:** Average of IC_50_, and Fold Change of the Synthesized Nanoparticles and Their Free Counterparts Using HepG2, Huh-7, and A549 Cancer Cells

Therapeutic Groups	HepG2 cells			Huh-7 cells			A549 cells		
	IC_50_,, µM (DOX, AV) and nM (CR)								
	IC_50,_ Mean	^1st ^FC	^2nd^ FC	IC_50_, Mean	^1st^ FC	^2nd ^FC	IC_50_, Mean	^1st^ FC	^2nd^ FC
DOX (Positive standard)	>100	Ref.	-	>100	Ref.	-	>100	Ref.	-
AV	>100	-	Ref.	>100	-	Ref.	>100	-	Ref.
DOX+AV	44.9 ± 3.61	2.22	2.22	>100	-	-	>100	-	-
AV NP	29.64 ± 4.01	3.37	3.37	32.5 ± 1.92	3.07	3.07	17.9 ± 0.91	5.58	5.58
DOX+AVNP	30.5 ± 2.18	3.27	3.27	31.6 ± 3.81	3.16	3.16	21.07 ± 2.27	4.74	4.74
CR	>100	-	Ref.	>100	-	Ref.	>100	-	Ref.
AVCR	>100	-	-	>100	-	-	>100	-	-
CR NP	46.12 ± 5.92	2.16	2.16	>100	-	-	>100	-	-
AVCRNP	35.4 ± 1.9	2.82	2.82	30.1 ± 2.65	3.32	3.32	18.3 ± 0.89	5.46	5.46
DOX+CRNP	36.7 ± 3.81	2.72	2.72	27.1 ± 1.43	3.69	3.69	18.2 ± 0.091	5.49	5.49

^1st^ FC, represents the fold change of the IC_50_, of each therapeutic group when taking DOX (the positive standard drug) as a reference (i.e. the IC_50_, of DOX divided by the IC_50_, of each group individually); ^2nd^ FC, represents the fold change of the IC_50_, of AV-based or CR-based therapeutic regimens when taking free AV or CR as a reference, respectively (i.e. the IC_50_, of free AV or CR divided by theIC_50_, of each AV-based or CR-based group individually); AV, avastin (Bevacizumab); DOX, doxorubicin; AV NPs, avastin (Bevacizumab) nanoparticles; CR, CCR2 inhibitor; CR NPs, CCR2 inhibitor nanoparticles; AVCR NPs, avastin-CCR2 inhibitor nanoparticles.

**Figure 2:**
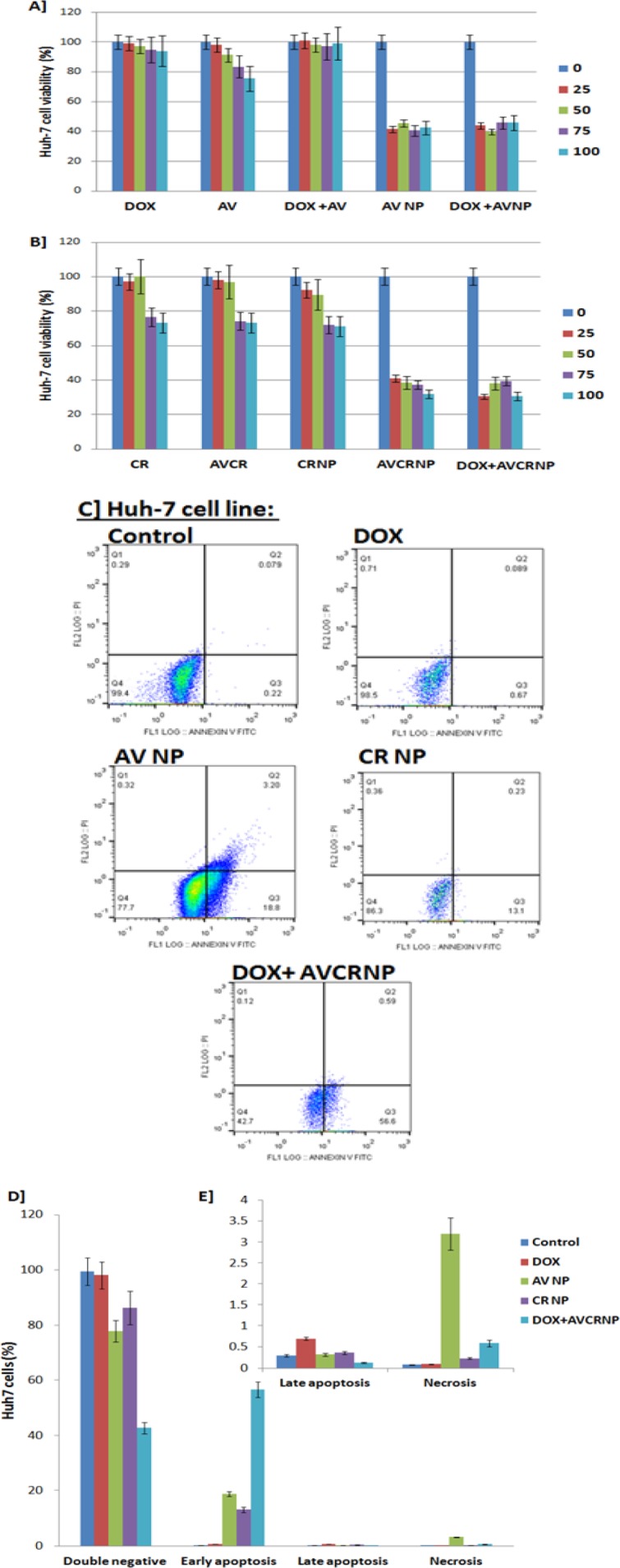
The Cytotoxic and Apoptotic Effects of the AV and CR Therapeutic Regimens against Huh-7 Cancer Cell Line. A, Huh-7 cell viability (%) upon DOX, AV, DOX+AV, AVNP, and DOX+AVNP treatments (n=3); B, Huh-7 cell viability (%) upon CR, AVCR, CRNP, AVCRNP, and DOX+AVCRNP treatments (n=3). The doses used for cytotoxicity were 0, 25, 50, 75, and 100 (µM for AV and DOX, and nM for CR); C, Representative images of Huh-7 cell apoptosis (annexin versus PI) using flow cytometry upon DOX, AVNP, CRNP, and DOX+AVCRNP treatments; D, E, Mean and standard error (n=3) of Huh-7 cancer cell early and late apoptosis, as well as necrosis versus control upon DOX, AVNP, CRNP, and DOX+AVCRNP treatments. The used dose for the flow cytometry experiments was either the IC_50_ dose for the drugs which recorded IC_50_ or 100 µM for DOX.The cells were incubated with the drugs for 24 h

**Figure 3:**
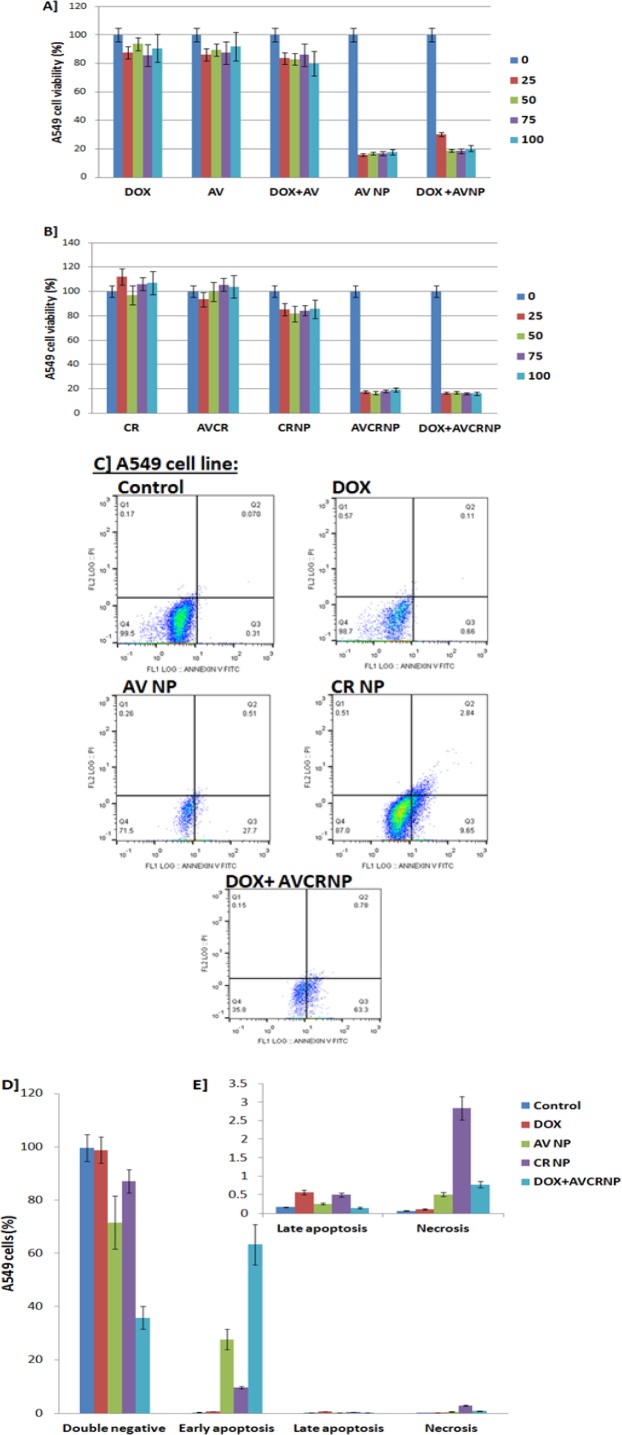
The Cytotoxic and Apoptotic Effects of the AV and CR Therapeutic Regimens against A549 Cancer Cell Line. A, A549 cell viability (%) upon DOX, AV, DOX+AV, AVNP, and DOX+AVNP treatments (n=3); B, A549 cell viability (%) upon CR, AVCR, CRNP, AVCRNP, and DOX+AVCRNP treatments (n=3). The doses used for cytotoxicity were 0, 25, 50, 75, and 100 (µM for AV and DOX, and nM for CR); C, Representative images of A549 cell apoptosis (annexin versus PI) using flow cytometry upon DOX, AVNP, CRNP, and DOX+AVCRNP treatments; D, E, Mean and standard error (n=3) of A549 cancer cell early and late apoptosis, as well as necrosis versus control upon DOX, AVNP, CRNP, and DOX+AVCRNP treatments. The used dose for the flow cytometry experiments was either the IC_50_ dose for the drugs which recorded IC_50_ or 100 µM for DOX.The cells were incubated with the drugs for 24 h

**Figure 4:**
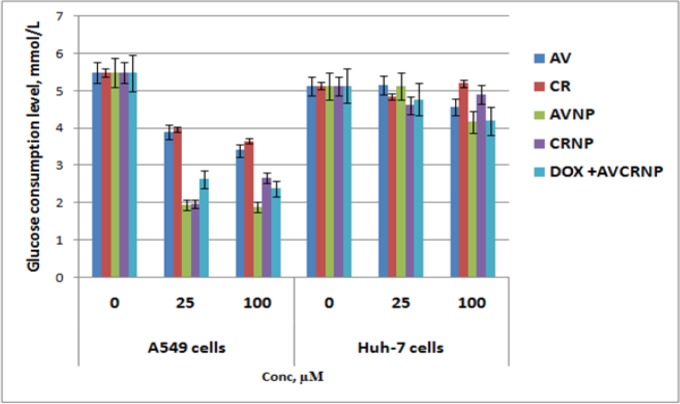
The Glucose Consumption Rate of A549 and Huh-7 Cancer Cells upon AV, CR, AVNP, CRNP, and DOX+AVCRNP. Mean and standard error (n=3) were represented in the blot. The used concentrations over cells were as follow: 0, 25, and 100 (µM for AV and DOX, and nM for CR). The cells were incubated with the drugs for 24 h

**Figure 5:**
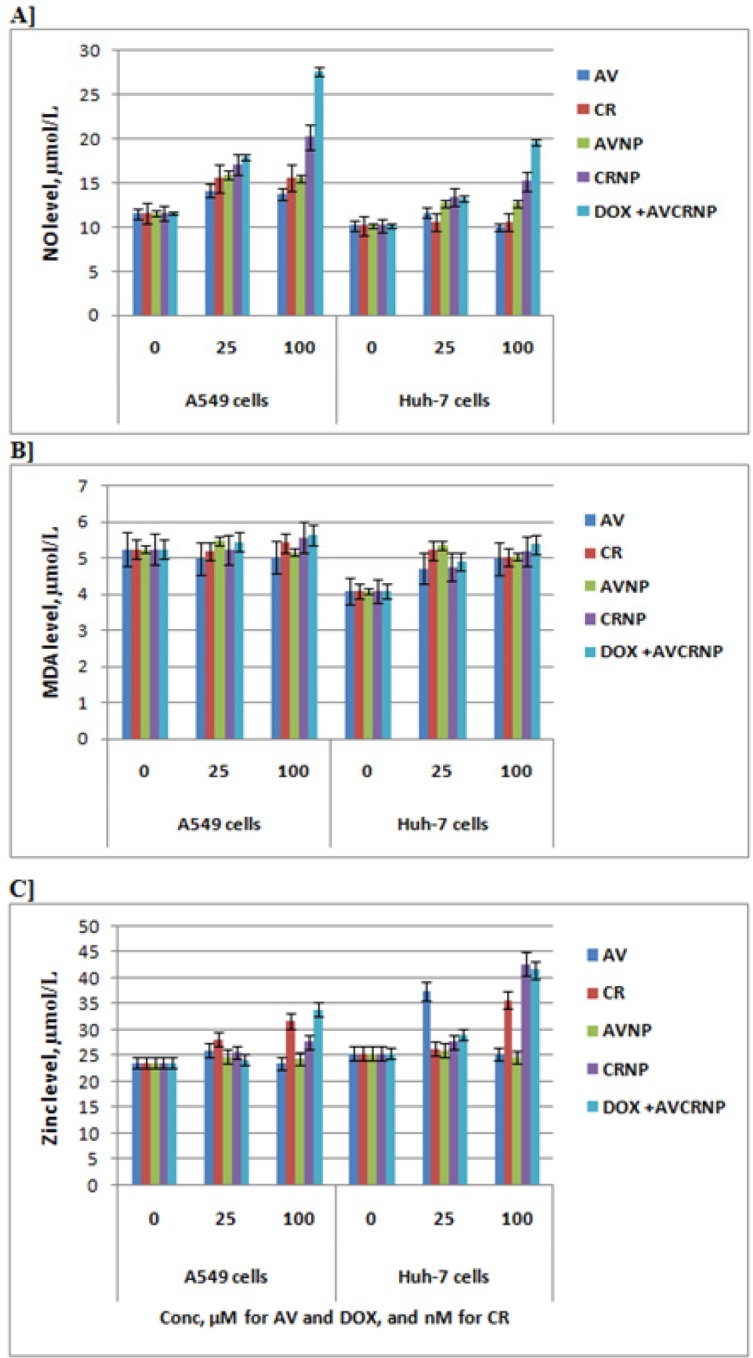
The Nitric Oxide (NO), Malondialdehyde (MDA), and Zinc (Zn) Levels of A549 and Huh-7 Cancer Cells upon AV, CR, AVNP, CRNP, and DOX+AVCRNP. Mean and standard error (n=3) were represented in the blot. The used concentrations over cells were as follow: 0, 25, and 100 (µM for AV and DOX, and nM for CR). The cells were incubated with the drugs for 24 h

*Nitric oxide assay*

Nitric oxide (NO) is rapidly oxidized to nitrite and nitrate which are used to quantitate NO production. Briefly, the cells were cultured in 96-well plates at a density of 1×10^4^ cells/well. In the second day, different nano-treatments and their free counterparts were added in the media. NO assay colorimetric kit was used to measure the total nitrate/nitrite in a simple two-step process Nitrate reductase was firstly used to converts nitrate to nitrite. Then, Griess reagent was used to convert nitrite to a deep purple azo compound. The amount of the azochromophore accurately reflected nitric oxide amount in the samples. Finally, optical density was measured at A540 nm using the microplate reader (BMG Labtech, Germany).

*Malondialdehyde (MDA) assay*

Quantification of lipid peroxidation is essential to assess oxidative stress in pathophysiological processes, where the end product of this process is malondialdehyde (MDA). Lipid peroxidation colorimetric assay kit is a sensitive detection tool of MDA. Briefly, the cells were cultured in 96-well plates at a density of 1×10^4^ cells/well. In the second day, different nano-treatments and their free counterparts were added in the media. The free MDA present in the sample was reacted with thiobarbituric acid (TBA) to generate a MDA-TBA adduct, which can be easily quantified colorimetrically at A532 nm using the microplate reader (BMG Labtech, Germany).

*Zinc levels measurement*

Zinc was quantified using a convenient colorimetric assay. Briefly, the cells were cultured in 96-well plates at a density of 1×10^4^ cells/well. In the second day, different nano-treatments and their free counterparts were added in the media. Practically, Zn was bound to a ligand and the colored product was then detected at an absorbance of A560 nm using the microplate reader (BMG Labtech, Germany). 

*Statistical analysis*

All assays were repeated three independent times (n=3). Comparisons between nano-formulations and their free counterparts versus controls were made using a two-tailed Student’s t test, and values of P < 0.05 were considered statistically significant.

## Results

*Characterization of nanoparticles*

The calibration curves of AV and CR after dialysis tubing were performed. The serial dilutions used for drawing the AV calibration curve were as follow: 0.39, 0.78, 1.56, and 3.12 mg/mL and for the CR calibration curve were as follow: 0.0078, 0.0156, 0.0312, 0.0625, and 0.125 mg/mL. 

As shown in (Table 1), average size distribution, zeta potential, polydispersity index (PDI), and EE% of the synthesized nanoparticles were tabulated. For nano-void (NV), we found that the nano-formulation F2 had the lowest average nano-size (110.3 nm ± 2.04 nm), best stability (highly negative charged zeta potential around -15.8 mV ±2.10 mV), and the lowest PDI (0.04 ± 0.00). The average nano-size F2 of the AV NP, CR NP, and AVCR NP were as follow: 147.8 nm ±3.4 nm, 76.26 nm ±3.4 nm, and 293.4 nm ±9.17 nm, respectively. The latter three nano-formulations were very stable with negatively charged zeta potential on their surfaces as follow: -9.05 mV ±1.2 mV, -2.81 mV ±1.2 mV, and 4.71 mV ±2.5 mV and very low PDI records as follow: 0.01 ±0.00, 0.07 ±0.00, and 0.04 ±0.00. The entrapment efficiencies (EE) of AV in the nano-capsules (AV NPs and AVCR NPs) were as follow: 86% and 82%, respectively. The entrapment efficiencies (EE) of CR in the nano-capsules (CR NPs and AVCR NPs) were as follow: 74% and 75%, respectively.

*AVCR NP plus DOX induced HepG2 cytotoxicity-mediated apoptosis*

In Figure 1A, HepG2 cells were treated with DOX as a known standard drug against solid tumors using different drug concentration (0, 25, 50, 75, and 100; µM for AV and DOX, and nM for CR) and showed that there was a significant reduction (P< 0.01) in the percentage (%) of HepG2 cell proliferation. Less than 40% inhibition of the HepG2 cells was observed over all concentrations compared to the control (100%), recording an undetectable IC_50_ (IC_50_ > 100). The same cytotoxic pattern of DOX was recorded with AV (IC_50_ > 100). Intriguingly, the combination of DOX and AV achieved detectable IC_50_ (IC_50_= 44.9 ± 3.61) upon HepG2 cell line. Importantly, AVNP and its combination with DOX remarkably sensitize the HepG2 cells, killing approximately 60% of the liver cancer cells with detectable IC_50_s (IC_50_s= 29.64 ± 4.01 and 30.5 ± 2.18, respectively) as shown in (Table 2).

In Figure 1B and Table 2, around 40% HepG2 cancer cell death was observed in case of CR and AVCR, with undetectable IC_50_s (IC_50_ > 100). Intriguingly, CRNP, AVCRNP, and DOX+CRNP increased the HepG2 cytotoxicity to be reached around 60% of liver cancer cell death, recording detectable IC_50_s (IC_50_s=46.12 ± 5.92, 35.4 ± 1.9, and 36.7 ± 3.81; respectively).

In Table 2, when taking DOX (the positive standard drug against HepG2 cells) as a reference, AVNP and DOX+AVNP recorded the highest fold change (3.37 and 3.27 times; respectively), followed by AVCRNP (2.82 times), DOX+CRNP (2.72 times), DOX+AV (2.22 times), and CR NP (2.16 times). We called this fold change as the 1st FC. When taking free AV as a reference against HepG2 cells, AVNP and DOX+AVNP recorded the highest fold change (3.37 and 3.27 times; respectively), followed by DOX+AV (2.22 times). While, when taking free CR as a reference, AVCRNP and DOX+CRNP recorded the highest fold change (2.82 and 2.72 times; respectively), followed by CR NP (2.16 times). We called this fold change as the 2nd FC.

In Figure 1C, D, and E, HepG2 cancer cell apoptosis using flow cytometry came to confirm the cytotoxic effects of the used AV and CR therapeutic regimens mechanistically. DOX+AVCRNP made around half (48.5%) of the HepG2 cancer cell population shifted from the double negative quadrant (negative annexin/negative PI) to the positive annexin/negative PI quadrant, causing early apoptosis with limited record for late apoptosis (negative annexin/positive PI) and necrosis (positive annexin/positive PI). 

DOX+AVCRNP was followed by AVNP in regard to HepG2 cancer cell death-mediated early apoptosis, where AVNP shifted around 20% of the HepG2 cancer cell population from the double negative quadrant to the positive annexin/negative PI quadrant, causing early apoptosis with limited record for late apoptosis and 3.5% of the cell population shifted to positive annexin/positive PI quadrant, causing some necrosis. In addition, CRNP and DOX caused 14.6 and 9.3% early apoptosis, as well as 3.9 and 2.7% necrosis, respectively, with limited late apoptosis as shown in (Figure 1C, D, and E). 

*AVCR NP plus DOX induced Huh-7 cytotoxicity-mediated apoptosis more than HepG2 cells*

In Figure 2A, Huh-7 cell line was treated with DOX as a known positive control drug against solid tumors using different drug concentration (0, 25, 50, 75, and 100 µM/nM) and showed that there was a non-significant gradual reduction (P> 0.05) in the percentage (%) of Huh-7 cell proliferation. Less than 10% inhibition of the Huh-7 cells was observed over all concentrations compared to the control (100%), recording an undetectable IC_50_ (IC_50_ > 100). The same cytotoxic pattern of DOX was recorded with AV but with a bit more inhibition in the Huh-7 cell proliferation, reaching over 20% with an undetectable IC_50_ (IC_50_ > 100). Opposite to HepG2 cells, the combination of DOX and AV achieved undetectable IC_50_ (IC_50_ > 100) with no mentioned inhibition of Huh-7 cells compared to control. Like HepG2 cells, AV NP and its combination with DOX remarkably sensitize the Huh-7 cells, killing approximately 60% of the liver cancer cells with detectable IC_50_s (IC_50_s= 32.5 ± 1.92 and 31.6 ± 3.81, respectively) as shown in (Table 2).

CR, AVCR, and CRNP killed around 25% of Huh-7 cancer cells at 75 and 100 µM doses, with undetectable IC_50_s (IC_50_ > 100). Intriguingly, AVCRNP and DOX+AVCRNP increased the Huh-7 cytotoxicity to be reached more than 60% of liver cancer cell death, recording detectable IC_50_s (IC_50_s=30.1 ± 2.65, and 27.1 ±1.43; respectively) as shown in (Figure 2B and Table 2).

In Table 2, when taking DOX (the positive standard drug against Huh-7 cells) as a reference, AVNP, DOX+AVNP, AVCRNP, and DOX+AVCRNP recorded high fold changes (3.07, 3.16, 3.32, and 3.69 times; respectively). We called this fold change as the 1st FC. When taking free AV as a reference against Huh-7 cells, DOX+AVNP recorded the highest fold change (3.16 times), followed by AVNP (3.07 times). While, when taking free CR as a reference, DOX+AVCRNP recorded the highest fold change (3.69 times), followed by AVCRNP (3.32 times). We called this fold change as the 2nd FC.

In Figure 2C, D, and E, Huh-7 cancer cell apoptosis using flow cytometry came to confirm the cytotoxic effects of the used AV and CR therapeutic regimens mechanistically. Huh-7 cancer cell was more sensitive than HepG2 cells to DOX+AVCRNP, where it made more than half (56.6%) of the Huh-7 cancer cell population shifted from the double negative quadrant (negative annexin/negative PI) to the positive annexin/negative PI quadrant, causing early apoptosis with limited record for late apoptosis (negative annexin/positive PI) and necrosis (positive annexin/positive PI). DOX+AVCRNP was followed by AVNP in regard to Huh-7 cancer cell death-mediated apoptosis, where AVNP shifted around 20% of the Huh-7 cancer cell population from the double negative quadrant to the positive annexin/negative PI quadrant, causing early apoptosis with limited record for late apoptosis and 3.2% shifted to positive annexin/positive PI quadrant, causing some necrosis. In addition, CRNP and DOX caused 13.1 and 0.6% early apoptosis, respectively. The latter had limited record for necrosis and late apoptosis. 

*AVCR NP plus DOX induced A549 cytotoxicity-mediated apoptosis more than Huh-7 and HepG2 cells*

In Figure 3A, A549 non-small cell lung cancer was treated with DOX as a known positive control drug against solid tumors using different drug concentration (0, 25, 50, 75, and 100 µM/nM) and showed that there was a non-significant gradual reduction (P> 0.05) in the percentage (%) of A549 cell proliferation. Around 10% inhibition of the A549 cells was observed over all concentrations compared to the control (100%), recording an undetectable IC_50_ (IC_50_ > 100). The same cytotoxic pattern of DOX was recorded with AV, observing an undetectable IC_50_ (IC_50_ > 100). Opposite to HepG2 cells, the combination of DOX and AV achieved undetectable IC_50_ (IC_50_ > 100) with no more cell inhibition compared to control. More than HepG2 and Huh-7 cells, AV NP and its combination with DOX remarkably sensitize the A549 cells, killing more than 80% of the non-small cell lung cancer with detectable IC_50_s (IC_50_s= 17.9 ± 0.91 and 21.07 ± 2.27, respectively) as shown in (Table 2).

In Figure 3B, CR and AVCR showed no cytotoxic effects against A549 cell line, but CRNP killed around 10% of A549 cancer cell line, with undetectable IC_50_s (IC_50_ > 100). Intriguingly, AVCRNP and DOX+AVCRNP increased the A549 cytotoxicity to be reached more than 80% of lung cancer cell death, recording detectable IC_50_s (IC_50_s= 18.3 ± 0.89 and 18.2 ± 0.091; respectively) better than HepG2 and Huh-7 cell lines. 

In Table 2, when taking DOX (the positive standard drug against A549 cells) as a reference, AVNP, DOX+AVNP, AVCRNP, and DOX+AVCRNP recorded high fold changes (5.58, 4.74, 5.46, and 5.49 times; respectively). We called this fold change as the 1st FC. When taking free AV as a reference against A549 cells, AVNP recorded the highest fold change (5.58 times), followed by DOX+AVNP (4.74 times). While, when taking free CR as a reference, AVCRNP and DOX+AVCRNP recorded slight difference in their fold changes (5.46, and 5.49 times; respectively). We called this fold change as the 2nd FC.

A549 non-small cell lung cancer apoptosis using flow cytometry came to confirm the cytotoxic effects of the used AV and CR therapeutic regimens mechanistically. A549 cancer cell was more sensitive than HepG2 and Huh-7 cells to DOX+AVCRNP, where it made 63.3% of the Huh-7 cancer cell population shifted from the double negative quadrant (negative annexin/negative PI) to the positive annexin/negative PI quadrant, causing early apoptosis with limited record for late apoptosis (negative annexin/positive PI) and necrosis (positive annexin/positive PI). DOX+AVCRNP was followed by AVNP in regard to A549 cancer cell death-mediated apoptosis, where AVNP shifted around 27.7% of the A549 cancer cell population from the double negative quadrant to the positive annexin/negative PI quadrant, causing early apoptosis with limited record for late apoptosis and necrosis. In addition, CRNP and DOX caused 9.6 and 0.66% early apoptosis, as well as 2.8 and 0.11% necrosis, respectively as shown in (Figure 3C, D, and E).

*AVCRNP plus DOX suppressed the A549 and Huh-7 glucose-based bioenergetics*

Figure 4 illustrates the glucose consumption rate from Huh-7 and A549 cell lines after our synthesized formulations therapy compared to control after 24 h incubation with the tested nano-drugs and their free counterparts. In the control, the glucose transporters worked in a sufficient way in all cancer cell lines and the cellular glucose uptake from the media increased significantly over 24 h, resulting in a significant decrease in the glucose levels remained in the media over the cancer cells (P<0.01). After that, the transporters slightly inhibited using the nano-drugs and their free counterparts, resulting in decreasing the ability of glucose passage from the media into the cancer cells over 24 h drug incubation, and in turn increasing the levels of glucose in the media compared to the control. 

*AVCRNP plus DOX induced A549 and Huh-7 cell reactive oxygen species (ROS)-stimulated apoptosis*

In Figure 5A, B, and C, we selected Huh-7 cancer cells as a model for hepatocellular carcinoma to test the redox status, because it was much sensitive against the used therapeutic regimens compared to HepG2 liver cancer cells. The redox status (NO, MDA, and zinc) of Huh-7 liver cancer cells versus A549 lung cancer cells were tested upon 0, 25, and 100 (µM for AV and DOX, and nM for CR) of AV, CR, AVNP, CRNP, and DOX+AVCRNP treatments. These order of treatments statistically increased (P> 0.05) the NO extracellular release of Huh-7 liver cancer cells and A549 lung cancer cells at 25 and 100 (µM for AV and DOX, and nM for CR), but the DOX+AVCRNP followed by CRNP statistically increased with higher significance (P< 0.01) the NO extracellular release of Huh-7 liver cancer cells and A549 lung cancer cells at 100 (µM for AV and DOX, and nM for CR). Knowing that, the latter increase was dramatic in the case of A549 lung cancer cells compared to Huh-7 liver cancer cells as shown in (Figure 5A).

For A549 lung cancer, there were no significant changes in lipid peroxide (malondialdehyde; MDA) levels after 25 and 100 (µM for AV and DOX, and nM for CR) treatments with AV, CR, AVNP, CRNP, and DOX+AVCRNP. On the contrary, there were significant increases of MDA production from Huh-7 liver cancer cells upon their treatments at 100 (µM for AV and DOX, and nM for CR) as shown in (Figure 5B).

Regarding zinc levels, it was increased in both cancer cells (A549 and Huh-7) after their tretments with CR and DOX+AVCRNP at 100 (µM for AV and DOX, and nM for CR). The nanoformulation of CR induced the elevation of zinc levels only in Huh-7 cells at the same concentration. For the latter cells, AV induced the production of zinc at 25 µM. At the latter concentration, there were no significant changes in the zinc levels released extracellulary from A549 lung cancer cell line as shown in (Figure 5C). 

## Discussion

There is an increasing evidence to the vital anti-proliferative effects of bevacizumab (avastin; AV) (Ferrara et al., 2005) and CCR2 antagonist (CR) (Monti et al., 2003; Wolf et al., 2012; Zhang et al., 2013; Zhang et al., 2010) , and recently their PLGA-based (Hao et al., 2009; Pan et al., 2011) and micelles-based (Roblek et al., 2015) nanoparticulates (NPs) on different cancer cells. One of the primary cancer types is hepatic cancer (HCC) (Goldstraw et al., 2011; Yang, 2009) and non-small cell lung cancer (NSCLC) is one of the most organs affected by metastasis (Shivakumar et al., 2016; Sasaki et al., 2011) . 

Our results indicated that AVCR NP sensitized DOX-treated A549 lung cancer cell line, which is one of the NSCLC types. This sensitization was approached by cytotoxicity-mediated apoptosis, which triggered by either glycolytic inhibition-mediated bioenergetics suppression or reactive oxygen species (ROS)-stimulated apoptosis. 

In the current study, we designed, manufactured, and characterized AV and CR NPs based on the knowledge of cancer cell microenvironment, to understand the underlying mechanisms that may facilitate providing novel anticancer therapies using these strategies. We used polyethylene glycol (PEG) and poly-l-lysine (PLL) for manufacturing the AV, CR, and AVCR NPs. Poly-l-lysine (PLL) has an advantage of being hydro- and bio-degradable, and biocompatible (Umano et al., 2011), and so it is suitable as a drug delivery carrier. It has been reported that PLL-modified poly (lactic-co-glycolic acid) (PLGA) NPs showed significantly higher entrapment efficiency (EE%) than PLGA NPs (Tahara et al., 2010). PLGA, when combined with PLL and PEG, has a relatively rapid rate of hydrolysis and it could reduce systemic clearance rates and prolong circulation half-life (Bao et al., 2015). Therefore, we selected PLL and PEG for encapsulating AV and CR to achieve bioavailable and biocompatible NPs.

Authors found that PLGA-PLL-PEG NPs are considered as an efficient drug delivery system, enhancing anti-cancer efficacy via intrinsic apoptosis pathway (Bao et al., 2015). The latter observation is in parallel with our results regarding AV and/or CR-loaded PEG-PLL NPs as promising anti-cancer platforms against lung A549 and liver Huh-7 cancerous cell lines via induction of early apoptosis through NO/MDA production and glycolytic inhibition.

In addition, authors used PLGA-based NPs for anti-VEGF inhibitor (ganciclovir (Duvvuri et al., 2007) and dexamethasone acetate (Xu et al., 2007) encapsulation, and they neither reported toxicity signs nor significant inflammatory responses for periods of up to two months (Giordano et al., 1995). In parallel, we used PEG-PLL NPs for anti-VEGF inhibitor (AV) encapsulation and it as a void (without drug) neither reported cytotoxicity against cancer cell lines as shown in zero concentration point in cytotoxicity panels. 

Our AV PEG-PLL NPs noted high entrapment efficiency (EE= 86%) of AV with high stability based on the zeta potential negative charges (-9.05 ± 1.2) noticed on the surface of the synthesized NPs, then z-average diameter came to confirm that it is in the nano-range (147.8 ± 3.4) with PDI = 0.01. These observed results were in agreement with a previous study (Li et al., 2012) indicated that the synthesized AV PLGA NPs had a slightly higher loading of AV (EE= 90%) compared to our NPs (EE= 86%), but in their case the PLGA particles lost its uniformity, this was most likely owing to partial phase separation of PLGA polymer and AV. When authors added PEG to their NPs (PEG-PLGA), the new NPs were long-circulating. This is due to, unlike PLGA, PEG is hydrophilic and retains water. The hydrophilicity of PEG also facilitated dispensing the particles compared to PLGA. This resulted in a lower yield of the particles as the majority of the polymer was dissolved in the water phase during particle preparation (Li et al., 2012). These results are confirming the advantage of our usage for PEG plus PLL instead of PLGA in the current study.

On the other hand, another research team encapsulated AV using PLGA NPs and the EE was lower than ours by 41% (i.e. EE = 45%). The release of the encapsulated AV from the NPs could last four weeks life-time (Hao et al., 2009). Pan and his companions also created long-lasting formulations, for a period of eight weeks, of AV through PEG-coated PLGA nanoparticles (Pan et al., 2011).

Authors tested a nano-based micelles approach for a targeted delivery of the CR to metastatic sites in lung. Blocking of CCR2 resulted in reduced tumor cell extravasation and inhibition of lung metastasis both in vitro (MC-38GFP cells) and in vivo models (Roblek al., 2015). The latter finding is in agreement with our synthesized CRNP which had a potential anti-apoptotic effect against lung A549 and liver HepG2 cancerous cells. 

The metastatic machinery initiation is strongly linked to the activation of the CCL2-CCR2 chemokine axis (Borsig t al., 2004; Cools-Lartigue et al., 2013; Gupta and Massague, 2006; Hiratsuka et al., 2013; Lu and Kang, 2009; Gul et al., 2014; Qian et al., 2011). Apparently CCR2-mediated signaling is involved at two levels: 1) recruitment of inflammatory monocytes to the pre-metastatic niche and 2) activation of endothelial CCR2 and thereby the induction of vascular permeability (Roblek al., 2015) . We suggest that the inhibition of the CCL2-CCR2 signaling axis during metastatic initiation will inhibit tumor cell extravasation and then metastases. We provided an in vitro evidence that CR NPs and AVCR NP enhanced the anti-proliferative effects of their free counterparts against human HCC (HepG2 cells) and NSCLC (A549 cells). In parallel, authors provided evidence that the monocyte-mediated trans-endothelial migration of both murine colorectal cancer cells and human melanoma cells in vitro is specifically inhibited by the CR. These results support the functional role of CCR2 signaling in the cross-talk among tumor cells, monocytes, and endothelial cells in this process (Roblek al., 2015). 

Importantly, these results indicate that local interference in the lung CCR2 signaling is sufficient for attenuation of metastasis. The idea of targeted drug delivery to desired tissues is not new. However, the application of a combinatorial therapeutic regimen (AV and CR) in a specific pegylated nano-composition has not been applied for anti-cancer approaches. We have used PEG-PLL as the carrier for delivering AV and CR to sites of liver and lung cancer cells. We showed that AVCR NP-targeting to cancer cells, causing cell death-mediated apoptosis, which was in line with previous observations (Roblek al., 2015). 

CCL2 has been previously targeted in various aliment cases, like liver cell damage and metastasis (Qian et al., 2011). Antibody-mediated depletion of CCL2 led to reduced immune cell infiltration to metastatic sites, while the use of CCL2 inhibitor reduced angiogenesis during liver fibrosis (Ehling et al., 2014). 

Mechanistically-wise, it was suggested that CCR2 regulates CCL2-induced breast cancer cell motility through MAPK- and Smad3-dependent mechanisms (Tangirala et al., 1997). Furthermore, authors examined the CCL2 expression in the culture media of different human NSCLC cell lines, and reported that A549 cell line secreted the highest amount of CCL2 and the expression level of CCR2 was the highest among all of the examined cells (An et al., 2017). Like our results, some authors indicated that the disruption of CCL2/CCR2 chemokine signaling has the ability to suppress A549 lung cancer cell proliferation and invasion (An et al., 2017). 

Since CCL2 is a chemokine with a wide range of features, the blockade of CCL2 may have unwanted defects. Therefore, we combined the CR with AV in a specific nano-platform tackling cancer cells and further experiments were performed to verify by which mechanism CCR2 antagonism inhibited A549 cell proliferation in vitro, and we founded that this strategy is depended on ROS-mediated early apoptosis. In addition, CR therapy reduced the CCL2- induced wound healing activity in A549 cells. However, CCL2 increased cell invasion by 4.2-fold (An et al., 2017). Together, our results suggested that CR inhibited CCL2-mediated A549 cell proliferation, induced apoptosis stimulated by ROS in vitro. The limitation of the CR treatment is the removal of it associated with an increased metastatic burden in an orthotropic mammary tumor model (Bonapace et al., 2014). On the contrary, the cessation of the CR treatment did not enhance cancer progression in the case of HCC postsurgical recurrence in vivo. Moreover, animals receiving the CR have a much longer survival rate (Li et al., 2017). 

The ERK1/ERK2 signaling pathway has been indicated as another mechanism in CCL2/CCR2-induced metastasis. (1) the association between CCL2/CCR2 axis and the MAPK pathways has been found in cervical, breast, and colon cancer cells (Gatti et al., 2017). (2) the expression of MMP-2 and MMP-9 can also be regulated by ERK1/2 in cancer cells (Kumar et al., 2010). (3) the direct link of MMP-9 regulation to CCL2/CCR2 axis was verified to involve the activation of Ras, ERK, and NF- κB pathway (Tang and Tsai, 2012). 

In conclusion, we and others found that targeting the chemokine receptor could inhibit cancer cell proliferation, migration, invasion, and induce ROS-mediated apoptosis. We found that AVCR NP sensitized DOX-treated A549 cancer cells more effectively than Huh-7 and HepG2 liver cancer cells. This inhibition of cancer cell proliferation was mediated by early apoptosis which stimulated by NO/MDA. Further in vitro and in vivo clarifications are required to understand the targeting mechanism of the CCL2/CCR2 pathway using CR and its nano-combination with AV in cancer therapy. Therefore, the CCR2 antagonist plus AV in their nano-formulation may be a potential novel therapeutic option in the treatment of CCR2-positive NSCLC and HCC.
